# Perceptions of laboratory animal veterinarians regarding institutional transparency

**DOI:** 10.1017/awf.2023.27

**Published:** 2023-03-17

**Authors:** Michael W Brunt, Daniel M Weary

**Affiliations:** Animal Welfare Program, Faculty of Food and Land Systems, The University of British Columbia, Vancouver, British Columbia Canada, V6T 1Z6

**Keywords:** animal experimentation, animal welfare, institutional openness, public communication, social licence, veterinarian attitudes

## Abstract

Institutions using animals for research typically have a veterinarian who is responsible for the veterinary care programme and compliance with regulatory obligations. These veterinarians operate at the interface between the institution’s animal research programme and senior management. Veterinarians have strong public trust and are well positioned to share information about animals used for scientific purposes, but their perspectives on sharing information with the public are not well documented and their perceptions of transparency may influence how institutional policies are developed and applied. The objective of our study was to analyse the perceptions of institutional transparency among laboratory animal veterinarians working at different universities. Semi-structured, open-ended interviews were used to describe perceptions of 16 attending veterinarians relating to animal research transparency. Three themes were drawn from the interviews: (i) reflections on transparency; (ii) reflections on culture; and (iii) reflections on self. Veterinarians reflected on their personal priorities regarding transparency and when combined with barriers to change within the institutions, sometimes resulted in reported inaction. For example, sometimes veterinarians chose not to pursue available opportunities for change at seemingly willing universities, while others had their initiatives for change blocked by more senior administrators. The sharing of information regarding the animals used for scientific purposes varied in how it was conceptualised by attending veterinarians: (i) true transparency; communication of information for the sake of openness; (ii) strategic transparency; attempt to educate people about animal research because then they will support it; (iii) agenda-driven transparency; selective release of positive stories to direct public opinion; and (iv) fearful non-transparency; not communicating any information for fear of negative opposition to animal research. Transparency was not perceived as an institutional priority by many of the veterinarians and a cohesive action plan to increase transparency that involves multiple universities was identified as a promising avenue to overcome existing barriers.

## Introduction

Research institutions sometimes avoid public communication concerning scientific experimentation on animals, and communications that do occur can include defensive responses relating to compliance with regulatory and welfare standards (Carbone 2021). Scientists perform experiments on animals under a social licence (Hughes 1958), an interwoven balance of authority, power, and trust between the scientists and society. Historically, this theory focused primarily on professional groups, but contemporary approaches apply social licence theory to industrial sectors and corporations. Institutions and people, who conduct animal research, occupy both professional and industrial aspects of social licence theory and must consider societal demands for transparency to maintain trust. Rollin (2004) built on aspects of social licence theory by recognising that society does not understand scientific practices with animals well enough to regulate them but expects scientists to self-regulate in ways that reflect societal values. Open communication builds public trust and maintains a social licence by demonstrating shared values (Arnot *et al.* 2016). However, claims of shared values (such as adherence to high welfare standards) without public access to evaluate claims, risks erosion of social licence (Hampton *et al.* 2020). Universities and other institutions conducting animal experimentation need to examine if their values align with those of the broader society in which they function, a process that can be aided by sharing information and remaining open to feedback (Carbone 2021).

Researchers increasingly understand the benefits of more transparent communication within the research community to improve reproducibility of scientific results, including the sharing of detailed methods and access to primary data (Landis *et al.* 2012; Percie du Sert *et al*. 2020; Cait *et al.* 2022); this sharing among researchers may also help maintain public trust in science (Yarborough 2014). Previous work has also shown that laboratory animal research that occurs in a more transparent environment improves perceptions of the research and the workers conducting it (Mills *et al.* 2018). However, even within an institution people vary in how they interpret the institutional culture of transparency about the use of animals (Brunt & Weary 2021).

Veterinarians responsible for animal care and compliance with regulations on animal use are important to study because they influence the development and enactment of institutional policies. Institutions using animals typically have one veterinarian with ultimate veterinary responsibility who reports to the senior administrator responsible for the animal care and use programme; for example, the ‘Attending Veterinarian’ in North America (National Research Council 2011; CALAM 2020), the ‘Designated Veterinarian’ in the European Union (European Parliament and the Council of the European Union 2010), and the ‘Named Veterinary Surgeon’ in the United Kingdom (‘Animals (Scientific Procedures) Act’ 1986). These positions are instrumental for an institution’s ‘Culture of Care’ that prioritises the commitment to improve the welfare of research animals (Ferrara *et al.* 2022). Recent publications extend the ‘Culture of Care’ to encompass research data quality and promotion of transparent communication (Brown *et al.* 2018; Robinson *et al.* 2020, 2022). However, how these veterinarians interpret and negotiate institutional transparency has not been assessed. Given the existing gap in the literature, the objective of the current study was to analyse the perceptions of institutional transparency among university veterinarians.

## Materials and methods

Participants were Attending Veterinarians (AVs) at Canadian universities. AVs were chosen because they exist at the interface between laboratory animal professionals (e.g. clinical veterinarians, animal technicians) and senior administrators. Full-time AVs from universities with a minimum of $C100 million of sponsored research income were chosen for recruitment as the complexities of these animal research programmes were thought to aid in the identification of diverse themes within the data. Twenty-one universities were identified as meeting the minimum sponsored research criteria (Research Infosource 2019). University webpages publicly identified and provided contact information for 13 AVs who were then recruited through criterion purposive sampling (Patton 1990). One AV was listed under a generic email address while seven universities did not publicly provide contact information for the AVs. Contact information for these AVs were obtained through snowball sampling (Patton 1990) or was previously known to the lead author (MWB) who had inside industry knowledge. Only 19 of the identified universities employed full-time AVs. Of these 19 individuals, 16 agreed to participate in the study and data saturation was achieved (Saumure & Given 2008). A series of demographic questions (age and gender-identity) thought to influence attitudes towards animal research were asked during participant recruitment (Pifer 1996; Hagelin *et al.* 2003). Eight participants identified as men, six as women, gender was not listed for one, and one preferred not to indicate gender identity. Two participants were within the age range of 30–39, five were 40–49, and nine were 50 or older. Five participants lived in Western Canada (British Columbia, Alberta, Saskatchewan, or Manitoba), nine in Central Canada (Ontario or Quebec), one in Eastern Canada (New Brunswick, Prince Edward Island, Nova Scotia, or Newfoundland), and one preferred not to indicate region of residence.

Semi-structured, open-ended interviews were conducted by MWB. The interview guide (available online at the Scholars Portal Dataverse: https://doi.org/10.5683/SP3/PYC4RY) was based on previous research (Brunt & Weary 2021). Participants were asked to describe their perceptions and experiences relating to animal research transparency at their institution. The first interview took place in person during February 2020. Logistical challenges surrounding the global COVID-19 pandemic delayed further data collection and the remaining fifteen interviews occurred over Zoom (version 5.4.2, Zoom Video Communications Inc) between November 2020 and March 2021. Research suggests Zoom is a highly suitable option for collecting interview data over geographically dispersed regions (Archibald *et al.* 2019). Interviews lasted between 47 and 90 min, with an average of 65 min. All interviews were audio recorded in Audacity (version 2.4.2, Audacity) and these recordings were then transcribed *verbatim* by an external company. As a means of transcript validation MWB listened to all interviews while reading the corresponding transcripts. Additionally, participants had an opportunity to review and change their interview transcript to more accurately represent their views (Lincoln & Guba 1985; Doyle 2007). This project was approved by The University of British Columbia’s Behavioural Research Ethics Board (H18-03395).

Applied thematic analysis was used to analyse transcripts (Guest *et al.* 2012a). MWB initially coded four interviews (NVivo, version 12.7.0, QSR International Pty Ltd). Iterative analysis across these interviews produced codes which emerged through comparison and axial coding (Charmaz 2006). Formation of the codebook occurred as parent codes were organised into themes. A second researcher trained in qualitative methods independently coded a subset of the data to establish inter-coder reliability and codebook validity (Guest *et al.* 2012b). Disagreements regarding code application were discussed until consensus was obtained. All interviews were then coded by MWB with the final codebook (available online at the Scholars Portal Dataverse: https://doi.org/10.5683/SP3/PYC4RY). When quotations were selected to illustrate a theme or code, the participant was identified with a randomly generated gender specific pseudonym (e.g. ‘Nelson’) and, if any additional words were added for clarity these were indicated with square brackets around the words.

## Results and discussion

Three themes were drawn from the data: (i) reflections on transparency; (ii) reflections on culture; and (iii) reflections on self.

### Reflections on transparency

This theme describes how AVs defined transparency. Some of these definitions included trust, confidentiality, connection, and empowerment. For example, Nelson described how transparency was linked to institutional accountability and acknowledgement of processes and activities that occur within an institution:

*“It’s being open with what is actually going on, what the policies are, the day-to-day level of actual work that is occurring at an institution. But being open and honest about following the policies and procedures as written. Rather than being inconsistent or kind of willy nilly where some people might have more favoritism than others. As an overarching aspect for all organisations, that’s the idea of transparency to me.”*

Some AVs attempted to contextualise different levels of transparency depending on who needed to know what information. For these meanings, transparency was not about being completely open but varied depending on the audience. Wes explained that an integral component of transparency involved:

*“The correct people having the correct information and level of detail that is most appropriate for them to understand animal research and animal use at this university. So there are different levels of transparency… There’s a level of detail that the animal use community needs, which maybe is different than the rest of the university community, which is different than the rest of the local community and the world beyond that. It’s making sure the correct people have access to suitable information to help them understand and make ethical and moral judgements* [about animal use]*.”*

However, other AVs defined transparency in a more dichotomous sense as either open or closed. Margo described her meaning of transparency and illustrated with an example of the relative obscurity of an animal facility at her institution:

*“Transparency is, how open are you. Are you readily telling the public where all your facilities are, what departments are working with animals, the species that are kept in your facilities? People who have worked in* [the same building] *where we have a gigantic animal facility, were not even aware that it was there. To me transparency is ‘are you allowing the public to see all that going on.’ I think that we have not been overly transparent, to be honest with you.”*

Charlie had a particularly difficult time with their description:

*“That’s probably the most difficult question that you put in front of me because it’s not something that I often thought about or tried to define in my mind. It’s not something I really thought about on a daily basis. I don’t because I’m busy in other areas. It’s also an uncomfortable area for me. I guess it means both visibility and openness.”*

In these four examples, Nelson, Wes, Margo, and Charlie defined institutional transparency through the clarity in which internal information or processes were communicated; this perspective fits well with other definitions of transparency (Hood 2006). Nelson, Margo, and Charlie described information that can be seen outside of the university while Wes added that the content and level of detail can vary dependant on the audience. These views are consistent with the concept that transparency is situationally contextualised and is not always translated into critical, logical, and rational thought (Worthy 2018).

Transparency also represented opportunities for discussion and learning from diverse perspectives. Randall described how the culture fostered by open and respectful dialogue promotes reflection across scientific disciplines:

*“An educational piece which then leads to discussion. So my perception is that animal protection groups play a very valuable role in the discussion because they allow us to reflect on what we’re doing from someone else’s perspective, potentially allowing us to make changes to what we’re doing in an informed way. Academic institutions should be based on free and open collegial discussion that then informs how we approach what we’re doing… Transparency ties into reproducibility and isn’t an issue that is unique to the use of animals in research. This is across the board in science. And, so to speak, transparency could be broadened to science as a whole. And this big black box of science needs to be opened up.”*

Other AVs expressed a more limited description of institutional transparency as a mechanism to control information, correct misunderstanding, or influence opinion about the use of animals in science. Abe exemplified this perspective by describing how the provision of information could be used to control understanding:

*“We’re not able to control the narrative, by not being transparent. Let’s be proactive in doing this and look at others that have been more proactively transparent. We do want to do it in a controlled balance way where we don’t get misrepresented or make ourselves targets* [for animal activism]*… Where are we getting misrepresented and want to really tell our side of the story? Share the evidence to support* [our side]*.”*

Abe’s description illustrated strategic transparency with an attempt to educate people about animal research to garner support. This perspective is consistent with research that found the animal research community expects that increased information will counter misinformation and public misunderstanding of its work (McLeod & Hobson-West 2016). Other AVs described a form of agenda-driven transparency to direct public opinion. This perspective has been attempted by some institutions who wish to increase support for their use of animals (Wadman 2017; Grimm 2018; Sanchez *et al.* 2018), by highlighting the benefits of animal-based research with curated information. The belief that such information is effective in leading to attitude change has been largely discredited in the public understanding of science literature (Wynne 1993). However, the idea that providing information will improve support continues to be popular (Wynne 2006) and remains prevalent in institutional science communication culture (Simis *et al.* 2016).

In contrast, Randall described true transparency; a process that fosters engagement with a diversity of perspectives, ultimately to inform institutional decisions. Similar sentiments were expressed by managers of animal research facilities (Brunt & Weary 2021). A collaborative discussion allows for diverse groups to participate and contribute in ways they deem appropriate (Carbone 2021). While research on mechanisms of transparency found that relatively few members of the public access available information (Worthy & Hazell 2017), those who choose to engage expect openness and a potential for change (Raman *et al.* 2018); an expectation not easily accomplished within dominant laboratory animal research governance structures (McLeod & Hartley 2017).

### Reflections on culture

This theme illustrates how AVs view the normative institutional values, beliefs, and conventions that guided the reported actions of people in the AVs’ university. This theme provides a referenced context to interpret actions (or lack thereof) of individuals or groups within that university relating to the sharing of information about animals used for scientific purposes.

### Institutional context

The accounts of AVs often described their institution as adverse to change. Here, Iris described how her university’s ethos reinforced the *status quo* even beyond animal research:

*“I think it’s generally the university’s culture. The culture of* [my university] *is very conservative. The Associate Vice President is very conservative* [and] *follows the standard line for a conservative person at a conservative institution. It’s generally speaking across all programmes, and it’s not just research with animals. The first thing* [my university] *does is see what other people are doing. They are not a leader in risk-taking at all. They’ll look and see what everybody else is doing first, before they’ll decide to stick their neck out. Especially when it comes to animal-based research… So even in a situation where there is a university at the pointy tip of the* [transparency] *spear, we don’t have the impetus to follow suit.”*

AVs also reported that legacies of informal institutional practices influenced discussions about animal research. Wes described influential senior administrators who created *“unwritten rules”* that, even long after their departure, reinforced a narrative that *“the university doesn’t talk about animals in research.”* Some AVs stated there was an institutional discomfort related to the use of specific animal species (*“dogs”* [Andy], *“cats”* [Dan], or *“non-human primates”* [Courtney]) or particularly invasive research on animals. Rachel recounted how a culture of secrecy formed when her university began research on non-human primates:

*“In the beginning we had some* [new] *researchers and from their experiences* [at other universities]*, monkeys and research in academia were not spoken about. So it’s the culture that has formed here despite my* [differing] *philosophy. It’s gone so far that we wouldn’t say monkey or non-human primate in emails. We would call the monkey something else because emails are open to freedom of information* [requests]. *It isn’t completely right but that is what we’re doing here.”*

Most AVs described their universities as being less transparent than others, with institutional fears commonly described as a barrier to transparency. Florence listed some of the fears at her university:

*“The concerns are that it could draw attention and potentially cause safety risks for the investigators themselves. Plus, potentially having protesters outside of our animal facilities, vandalism on university property. Also, how that* [attention] *could sway the public perception of the university and the university’s reputation… It’s a matter of breaking down people’s fear about it. It’s always been taboo to talk about this stuff. Obviously it’s going to take some time; this notion has to grow on* [the senior administrators]*.”*

While Florence offered solutions that involved a slow evolution for the university’s transparency culture, other AVs highlighted a lack of institutional motivation to change. Joey stressed that in the absence of internal (strong advocates) or external (animal activism) pressures, transparency is not perceived as a priority:

*“I don’t know if there’s enough momentum on its own merits to do it… We haven’t gotten there on our own. In other words, just being intrinsically motivated, we’re not at the level of transparency that other institutions are… I think if you rely on each individual institution coming to their own conclusions and ultimately doing it for themselves, you’re going to wait for a very long time. Although, if it was someone’s goal to suggest that across Canada institutions of higher learning, government research facilities, and private industry* [publicly report] *exactly how many animals are used, how they’re used, and what the outcomes of that are; that would have to be a top-down effort in my view. It would have to be mandated by regulatory bodies or granting agencies. You’re not going to see it for a very long time if you’re looking for it to happen from a ground up scenario. It’s not because every institution is hiding itself in a cloak of protection but there are simply too many other competing priorities within those institutions to allow it. It doesn’t make it high enough in the food chain.”*

Descriptions of institutional cultures of transparency varied but as highlighted above focused on cautiousness and reluctance to lead change (Iris), unwritten rules (Wes and Rachel), fear (Florence), and inertia (Joey). These verbal accounts demonstrate the diversity of ways in which universities arrive at practices for sharing (or limiting) information about the use of animals in their research programmes.

### Influential roles

Multiple advisers were identified with authoritative perspectives; attending veterinarians (e.g. *“An independent voice”* [Kevin]), Animal Care Committees (e.g. “*A diversity of viewpoints from across the university community”* [Joey]), communications experts (e.g. *“Lay person insights”* [Randall]), and researchers (e.g. “[Their buy in] *would be a critical step”* [Florence]). AVs described these advisors as having varying levels of influence within their institution and sometimes contributed to tensions that hindered transparency. Even when all these advisors come together in a favourable environment, barriers still need to be overcome:

*“It’s about creating the culture. The key people that are passionate about this in the right roles; public affairs, Animal Care Committee, directors, academic programmes. They might not agree on how to get there but they agree that we should be getting there. Out of that comes ideas. Unfortunately for institutions, it is hit and miss on who the executives are. You could have four years of nothing because* [every] *time you bring it up* [they say], *‘There’s no way I’m going there in my tenure.’ But then you get some executives, who say, ‘Yeah, let’s try that.’ I think you’ll find that there’s more progressive executives in the world, as we progress”* [Bill].

Bill’s example illustrates the powerful role that senior administrators have within the governance structure, a point raised by many participants. AVs described that many decisions are made behind closed doors with a small group of executives. Dan described several unsuccessful initiatives and lamented, *“The big shots can shut this down or let us do it… All I want is the university not* [to] *block things.”* Similarly, other AVs described how changes to institutional policy can occur when decision makers are willing to take leadership on this issue. As Randall described:

*“Ultimately, it was the Vice President Research at the institution that brought* [my recommendations] *to the attention of the university’s senior leadership team… There were concerns that a major donor would see that we were being public about using animals and not want* [their] *funds to support the university’s mission. It didn’t go further than that discussion* [because of] *the foresight of our Vice President Research, who felt that* [animal research] *wasn’t something that we should be ashamed of and that this is a part of an academic institution, whether you like it or not. We proceeded with* [an increased] *level of transparency.*

While the AVs’ accounts focused on influential advisors within a university, the persuasive impact of senior administrators from other universities (e.g. *“*[They] *love to be associated with the* [elite group of] *Canadian research universities and would follow their lead”* [Andy]) and the power of regulators and granting agencies (e.g. “*Solve the problem with one stroke”* [Joey]) illustrated how external entities could shift institutional culture. Additionally, AVs highlighted the influential role the local community plays in prioritising transparency within universities (e.g. *“There’s no demand for transparency. We’re not* [a] *cosmopolitan* [city]*”* [Margo]). However, as Bill described, public ignorance regarding where animal research is occurring also plays a role:

*“Maybe I have been a little loose on the idea of* [the public] *not caring. It’s probably more they don’t know what they don’t know. They don’t know that walking down that busy street there’s a research facility in the basement of that building. They wouldn’t think to look at a website to find out how many animals are used year-over-year in the basement of that building. They don’t know what’s going on at the university. Which in itself is a bit of a problem. The vast majority* [of people] *don’t know what’s happening and can’t* [give input] *since* [they] *don’t know to look.”*

AVs identified numerous advisors within their institutional hierarchy and external roles that contribute perspectives that could influence university practices; decision-makers, AVs, directors, granting agencies, regulators, and the external university community. These results highlight that there are layers of management above AVs that impact how and if transparency is enacted.

### Decision and bureaucratic processes

While multiple internal and external agents are positioned to influence decisions, Dan, Bill, and Randall described decisions at their institutions are ultimately made by a small group. Having worked at several universities, Bill explained that while most processes involve a diversity of perspectives, decisions are often driven by considerations of risk to the university:

*“So you’ve got transparency and confidentiality swirling around every day. You* [also] *have universities that are terrified of risk to reputation… The risk to reputation is a big one and it’s driven by a very very small number of people with the ability to* [authorise] *use* [of] *institutional resources. The degree to which you actually are going to be transparent is driven mostly by the perception of a very small group of executives on what risk to reputation means. Even if everyone agrees, you could have two executives drive decisions based on* [their] *10-minute watercooler conversation. ‘You know, this scares me. Let’s not do that.’ That drives transparency and the lack of institutional resources. It sets a culture.”*

Barriers, such as the absence of an institutional transparency policy (e.g. *“There isn’t a clear delegation”* [Wes]) or competing academic interests (e.g. *“Don’t want to come across as advocates for animal research and say downplay animal rights philosophy”* [Randall]), were noted as bureaucratic road-blocks by AVs. Many accounts illustrated that the lack of institutional resources hampered the ability of AVs to change institutional transparency practices. Resources not only included personnel dedicated and responsible for advancing these areas but, also included *“time”* [Brooke] from busy AVs and executives. Here, Joey illustrates these challenges:

*“Senior administrators at my institution are really overscheduled. They don’t have enough time already. If I wanted to get something on their table and really get them to move on it, I’d have to present it to them as a package. I’d have to do the legwork, tie it up with a bow, drop it on their desk and say ‘This is what we need to do. Here’s how we would do it and this is what it would look like.’ I don’t have time for that. I maybe could have done it before I became the AV but at that time I was still earlier in my career and learning the art of laboratory animal medicine. It’s one of those incongruous things where you have more time earlier in your career but you don’t have the knowledge or the understanding. Once you develop it, you’ve got so many other things on your plate that you can’t go anywhere with it. It’s a conundrum.”*

In summary, this theme addresses the why, the who, and the how regarding the institutional culture of transparency at universities. Participants provided details about institutional contexts that influenced the development of this culture. Other research has identified that an intended aim of reduced transparency is to reduce reputational risk for institutions (Bennett & Ringach 2016). However, reduced transparency may instead lead to increased alienation of the public and mistrust in institutions (Wynne 2006). Many influential roles, occupying various degrees of prominence, were described inside and outside the interviewees’ universities. The ability of executives within the university to control the flow of information and institutional resources was often identified as a barrier to change. Most participants did not identify institutional transparency surrounding animal research as a priority for their university but believed it became so if influenced by internal and external pressures. Carbone (2021) challenged institutions to move beyond the current transparency models and instead pursue a more collaborative approach (including, for example, animal protection groups) increasing diversity of expertise and opinion. Inclusion of diverse opinions may lead to improved decisions and push the moral (Birchall 2011) and political (Birchall 2014) boundaries of transparency. Other authors have suggested that scientists using animals would benefit from the support of funding agencies and their academic institutions, including the development of transparency policies and financial support for initiatives that improve contact with the public (Ringach 2011; Bennett & Ringach 2016). We encourage institutions that use animals, and granting agencies that fund scientific research with animals, to have clear transparency policies for communication with the public.

### Reflections on self

This theme reports examples of where AVs used the interview to engage in self-reflection. The importance of this theme relates to its capacity to demonstrate how AVs reflect on their own actions and how these actions have reportedly influenced their university’s culture of transparency. While Randall identified himself as a change agent in increasing transparency at his university, he expressed frustration with the lack of progress from other universities:

*“It was really myself that was driving* [institutional transparency] *as the university veterinarian. I feel very strongly around transparency. I attended a two-day conference around discussions of transparency that was targeted to research intensive universities and hospitals across Canada. Unfortunately, it was very hard to get traction at other institutions. I was fortunate enough to have the backing of my university* [senior administrators] *and was still able to pursue it at a more local level.”*

Unlike Randall, Charlie reflected that unease with the potential unknown consequences of increased transparency had reinforced fearful non-transparency at their university:

*“If you talk to somebody else, they might say, ‘Well, that’s because of Charlie’s nature.’ That’s possible and I think those things all play together. It’s an area of discomfort about all of this. Because how much of it is really me and how much of it is the institutional climate because of the senior leadership? How much of it is because of the people in the community who are substantially different than people in other communities?… I see large scale transparency as opening myself up for more* [public questions] *that I don’t actually like to do. And I guess that’s probably where that reservation about transparency comes from, and why I tend to sway the argument in one direction rather than the other… I’m pretty content with the way things are done. That’s probably because I’ve influenced it that way and I’ve seen it be largely successful in this institution and in this community… So I’m sure it’s me and that drives some of the processes in our institution. I’m sure of it.”*

Many of the AVs reflected on how they personally prioritised transparency. Margo considered her role in maintaining her university’s low level of transparency:

*“I know the culture has not been very transparent. Present and past directors* [were] *not really keen on being transparent. Have I fallen into that? Maybe I’m admitting my own faults here? I think we could do it if we felt it was necessary.* [We] *haven’t been pushed to the point where we feel that we need to make some sort of grandiose gesture. Maybe I should be pushing* [transparency]*?”*

In summary, this theme illustrates the reflective capacity and the diversity of personal priority levels for transparency. The differences between AVs influenced perceived opportunities to enact initiatives, since information can be transformed and shaped by the biases and assumption of those who interpret it (Worthy 2018). Most self-reflections illustrated the variation in the personal priority placed on transparency by the AVs, and varying levels of institutional willingness for transparency. This combination could in some cases result in change, and in other cases inaction. In the latter case, this can be because AVs may have their initiatives blocked by senior administrators, or because the AVs did not pursue available opportunities at willing universities. Overwhelmingly, a unified approach was endorsed by interviewees that involved all universities increasing transparency in the same way at the same time; however, there was less agreement about what specific information and the purpose of the information provided to the public. The concept of creating accountability for public institutions through transparency is universally supported across political contexts but enactment of transparency is often debated (Birchall 2014).

### Limitations and future research

The qualitative nature of the current study limits our ability to generalise to the broader population. However, the inductive qualitative methodology provides a deep understanding of our participants’ specific perceptions of institutional transparency. Transference of this knowledge is encouraged in new studies to identify whether top-down (regulatory or granting agency mandates), bottom-up (voluntary transparency agreement between Canadian universities that use animals for scientific experiments), or a combination of both initiatives would meet the needs and expectations of academic institutions, animal researchers, laboratory animal professionals, and the multidimensional publics. Transparency and the purpose of openness are constructed differently by diverse groups (McLeod & Hobson-West 2016). Research on mechanisms of transparency found that relatively few members of the public access available information (Worthy & Hazell 2017), but that those who choose to engage expect openness and a legitimate potential for change (Raman *et al.* 2018). Supporting the belief that some publics desire information flow in a two-way dialogue rather than simply the provision of information.

A potential limitation of the current study is the absence of perspectives from the three (of 19) AVs approached who were not willing to participate. Two of the three individuals stated they were too busy to schedule an interview while the third said their experiences made them sceptical and cynical about institutional commitments to transparency and did not see value in the current study. Authors must consider if they have reached the full range of variation on a phenomenon given the absence of potential contributors (Groger *et al.* 1999). While the views of these three individuals would have aided our description of institutional transparency at large Canadian universities, the redundancy detected across the accounts of our 16 participants provides us with some confidence in data saturation. We encourage additional research to understand different perspectives (e.g. scientists, regulators, senor administrators), sectors (e.g. smaller universities, government, private industry), and geographical regions (e.g. USA, EU, UK, China).

Additionally, the current study focused on perceptions of AVs regarding institutional transparency related to the use of animals for scientific purposes. The regulatory, legal, and oversight systems that govern animal research vary internationally and mandate various degrees of information release, so individual institutions may not have complete autonomy regarding what information is released. Additional research is encouraged on the intersection of human participant research and animal subject research, specifically on how international ethical codes and broad decision-making principles for humans could inform policy for animal research.

### Animal welfare implications

Overall, our results highlighted the value of social science research to analyse complex concepts that integrate social values like transparency and animal welfare. Specifically, while AVs are charged with institutional programmes that oversee the welfare of research animals, the decisions regarding what is communicated to the broader public concerning the lived experiences of these animals is often determined by more senior administrators. Additional information shared with the public could improve decision-making through the incorporation of social values (Beauchamp & DeGrazia 2020). Understanding perspectives of minority opinions (Raman *et al.* 2018), including indigenous perspectives (Hudson *et al.* 2019), through diversified opportunities to participate in the animal research governance process can begin to address value-ladened issues like animal welfare. Two specific issues would benefit from further societal input: (i) what is the upper limit of suffering an animal should be allowed to endure for our benefit; and (ii) what research is sufficiently valuable to justify any level of suffering?

## Conclusion

The sharing of information regarding the animals used for scientific purposes is a complex topic with variation in how it is conceptualised by AVs, the influence of internal and external pressures, and perceived barriers to increased transparency. Transparency was conceptualised in four ways: (i) true transparency; communication of information for the sake of openness; (ii) strategic transparency; attempt to educate people about animal research because then they will support it; (iii) agenda-driven transparency; selective release of positive stories to direct public opinion; and (iv) fearful non-transparency; not communicating any information for fear of negative opposition to animal research. Given the diverse understanding of the purpose of transparency, we suggest that active and sustained communication between senior administrators, university veterinarians, animal care staff, and scientists is necessary to build a consensus on how to pursue transparency; the lack of such a plan to increase transparency was identified as an important barrier by attending veterinarians. While generating consensus at the local institutional level is important, substantial progress would likely benefit from involvement of professional associations (e.g. of senior administrators, laboratory animal professionals, scientists), regulators, and funding agencies to work collaboratively and agree to a shared vision of transparency.

## Data Availability

Data are stored at the Scholars Portal Dataverse: https://doi.org/10.5683/SP3/PYC4RY. Data are not publicly available due to concerns of participant confidentiality with transcripts in the public domain. Access to the underlying data can be requested from ubc.all-reb@ubc.ca at the Behavioural Research Ethics Board at the University of British Columbia.
